# The Implication of Land-Use/Land-Cover Change for the Declining Soil Erosion Risk in the Three Gorges Reservoir Region, China

**DOI:** 10.3390/ijerph16101856

**Published:** 2019-05-26

**Authors:** Jinzhu Jiu, Hongjuan Wu, Sen Li

**Affiliations:** 1 School of Environmental Science and Engineering, Huazhong University of Science and Technology, Wuhan 430074, China; jiujz@hust.edu.cn; 2 Centre for Ecology & Hydrology, Wallingford OX10 8BB, UK; 3 Environmental Change Institute, University of Oxford, Oxford OX1 3QY, UK

**Keywords:** soil erosion risk, Three Gorges Reservoir Region, RUSLE, land cover change, China

## Abstract

The Three Gorges Reservoir Region (TGRR) in China is an ecologically and politically important region experiencing rapid land use/cover changes and prone to many environment hazards related to soil erosion. In the present study, we: (1) estimated recent changes in the risk pattern of soil erosion in the TGRR, (2) analysed how the changes in soil erosion risks could be associated with land use and land cover change, and (3) examined whether the interactions between urbanisation and natural resource management practices may exert impacts on the risks. Our results indicated a declining trend of soil erosion risk from 14.7 × 10^6^ t in 2000 to 1.10 × 10^6^ t in 2015, with the most risky areas being in the central and north TGRR. Increase in the water surface of the Yangtze River (by 61.8%, as a consequence of water level rise following the construction of the Three Gorges Dam), was found to be negatively associated with soil erosion risk. Afforestation (with measured increase in forest extent by 690 km^2^ and improvement of NDVI by 8.2%) in the TGRR was associated with positive soil erosion risk mitigation. An interaction between urbanisation (urban extant increased by 300 km^2^) and vegetation diversification (decreased by 0.01) was identified, through which the effect of vegetation diversification on soil erosion risk was negative in areas having lower urbanisation rates only. Our results highlight the importance of prioritising cross-sectoral policies on soil conservation to balance the trade-offs between urbanisation and natural resource management.

## 1. Introduction

Soil erosion by water is one of the most sensitive factors shaping the pattern of land degradation [1]. Soil erosion can lead to reduced agricultural productivity, intensified the occurrence of flooding and ecological disasters [2], result in sediment accumulation in riverway [3] and cause water environment deterioration [4]. It is important to quantify the impacts of soil erosion by water in order to develop effective actions for soil and water conservation.

The underlying processes of soil erosion can be triggered by both natural and anthropogenic factors. Natural factors include precipitation, soil texture, terrain slope, and vegetation coverage [5]. Anthropogenic drivers induced by human include urbanisation, cultivation and land management. Urbanisation motivated rural labour to migrate to cities, which led to abandonment of croplands [6] and exerted a positive effect on soil conservation [7]. Deforestation, dryland farming and overgrazing can amplify soil erosion. Conversion from cropland to grassland helps to improve soil and water conservation capacity [1]. Vegetation diversity can also play an important role in mitigating soil erosion, which not only could contribute to healthy estuarine areas, but also help maintaining soil fertility on pasture land [8]. The ecological restoration policy in China, i.e., the Grain to Green Programme (GTGP), is reported to have a positive effect on soil conservation via afforestation [7]. While contemporary environment management is facing challenges to enable land development for different uses, balancing between urbanisation and natural resource protection has always been a key focus. It remains largely unclear whether and how interactions between urbanisation and natural resource protection might have an impact on soil erosion.

The Three Gorges Reservoir Region (TGRR), which stretches along the middle and upper reaches of the Yangtze River in China, contains the largest water conversation project in the world [9]. As an essential component of the Yangtze River basin, the TGRR possesses complex ecological and social-economic characteristics, and is prone to many environmental problems in relation to soil erosion [10]. As the majority of the region is rural, agricultural practices have been causing soil erosion and altering the carbon balances and feedbacks in soil [11]. Soil erosion is associated with downstream export of sediments, nutrients and pesticides, which affect the quality of surface water and increase the risk of flooding [12,13]. Soil erosion can cause degradation of habitats of important species, challenging the provisioning of ecosystem services [14]. Studies previously conducted in the TGRR mainly focused on small watersheds and estimated soil erosion risk using both field and modelling approaches, such as runoff plot observation, artificial simulated rainfall, erosion needle, nuclide tracer, Soil and Water Assessment Tool (SWAT) and the Water Erosion Prediction Project (WEPP) model [15,16,17]. Taking the Hubei section of the TGRR as the study area, Cai, et al. [18] found that slope was positively correlated with the risk of soil erosion, and purple soils experienced greater erosion. The sloping farmland was found to be the most seriously eroded land use type in the Lingtangjiao watershed of the TGRR [19]. On Wuling Mountain, the presence of vegetation was reported as an important factor of a lower soil erosion risk [5]. Yan, Wen, Shi, Ju and He [15] planted cash crops on the ridge to reduce soil erosion in Zhong County. There has been relatively little attention paid, however, to the monitoring and evaluation of soil erosion over the whole of the TGRR from a long-term perspective. Moreover, existing studies mainly focused on the influence of natural factors on soil erosion in the TGRR, the influences of anthropogenic factors are less understood, albeit land-use and land-cover are changing rapidly in this ecologically and politically important region in China.

The main objectives of this study are twofold: first, to estimate changes in the spatial risk patterns of actual soil erosion for the whole of TGRR between 2000 and 2015; second, to explore the consequences of land use/cover change on soil erosion, via identifying key land cover types associated with soil erosion change and investigating the potential effects of interactions between urbanisation and natural resource management on soil erosion change. The time period focused in this study allowed us to assess and compare the situations at present and prior to the first main generator of the Three Gorges Dam started to operate (i.e., in 2003 when the water level started to rise). Our study was built upon data compiled from multiple sources. A combination of analytical methods were applied, including the Revised Universal Soil Loss Equation (RULSE) [20], which is the most commonly used model to estimate long-term soil erosion rates in large-scale studies [21] and statistical regressive models. We intended to obtain results which could serve as a scientific basis and reference for integrated urban and rural planning and water and soil resource management in the TGRR.

## 2. Study Area

**Figure 1:**
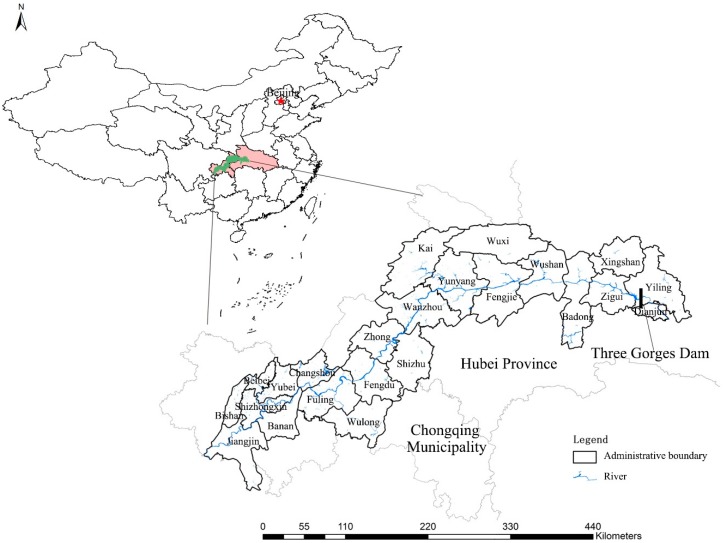
Study area: the Three Gorges Reservoir Region.

**Figure 2:**
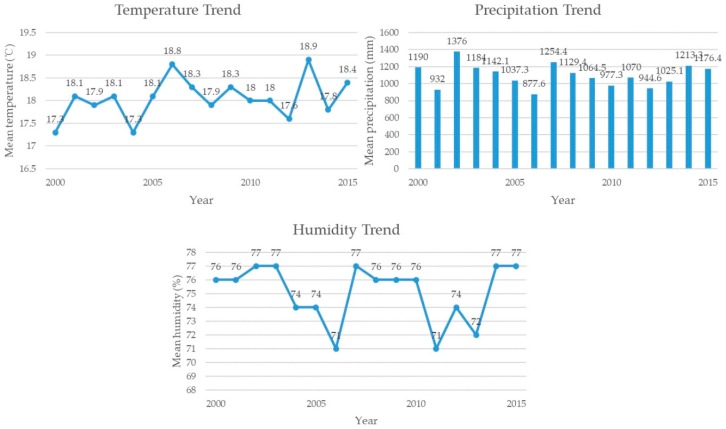
Temperature, precipitation and humidity trends from 2000 to 2015 in the TGRR.

The Three Gorges Reservoir Region (TGRR) is located between latitude 28°56′ N–31°44′ N and longitude 106°16′ E–111°28′ E covering the lower section of the upper reaches of the Yangtze River, with an area of 5.8 × 10^4^ km^2^ and with a population of 14.8 million (2016) [22]. It consists of 23 counties, county-level cities and prefectural districts of Chongqing Municipality (Wushan, Wuxi, Fengjie, Yunyang, Kaizhou, Wanzhou, Zhong, Shizhu, Fengdu, Wulong, Fuling, Changshou, Yubei, Banan, Jiangjin, Shizhongxin) and Hubei Province (Xingshan, Yiling, Dianjun, Zigui, Badong) (see Figure 1). Approximately 74% of the region is mountainous, 4.3% is plain area and 21.7% is hilly area [23]. The TGRR has a subtropical monsoon climate with dense and diverse vegetation [24]. From 2000 to 2015, the region’s annual average temperature increased from 17.3 to 18.9 °C, annual precipitation increased from 877.6 to 1376 mm and annual humidity increased from 71 to 77% (see Figure 2).

## 3. Materials and methods

### 3.1. Soil Erosion Risk Estimation

The Revised Universal Soil Loss Equation (RUSLE) is widely accepted as a simple, effective and explanatory tool to estimate risk of actual soil erosion at large scales [17,19,25,26]. In this study, while data were prepared at various spatial resolutions (Table 1), final soil erosion risk was estimated on a 1 km × 1 km cell grid to cover the whole of study area for four time periods: 2000, 2005, 2010 and 2015. Data manipulation and presentation were conducted using ArcGIS Desktop 10.6 (Esri, Redlands, CA, USA).

For each cell, the RUSLE estimated the annual actual soil erosion by water, A (t·km^−2^·y^−1^), as a product of six environmental factors:

*A* = *R* × *K* × *LS* × *C* × *P* (1)

where *R* is the rainfall-runoff erosivity factor (MJ·mm·km^−2^·h^−1^·y^−1^); *K* is soil erodibility factor (t·km·h·km^−2^·MJ^−1^·mm^−1^); *LS* is the slope length and steepness factor; *C* is the cover management factor; and *P* is the support practice factor.

#### 3.1.1. Rainfall Erosivity Factor (*R*)

**Figure 3:**
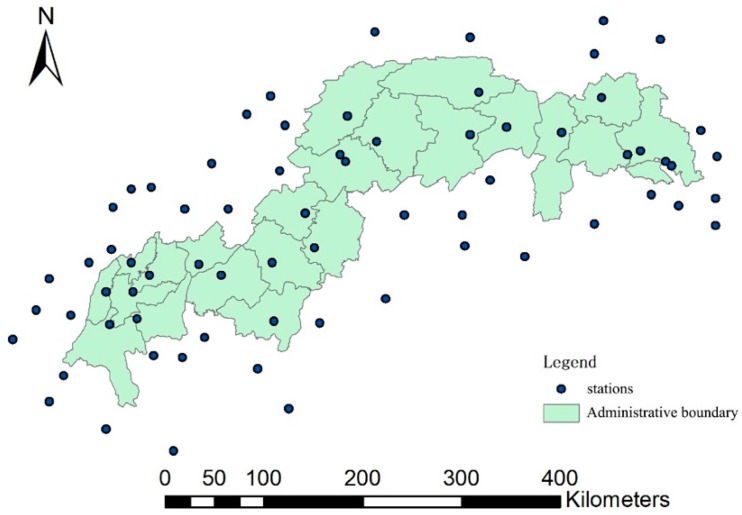
Location of meteorological stations used in this study across the TGRR.

*R* is an indicator of the capability of water to detach and transport soil particles. It is sensitive to the intensity and duration of rainfall. In this study, daily precipitation data purchased from the local meteorological stations were used to calculate *R*. To cover the whole and neighbouring area of TGRR, 69 stations were selected with a 60-kilometers buffer of the study area (Figure 3). The *R* factor was calculated following an approach widely used in China, for example by the National Water Conservancy Survey [27,28]:

(2) $$R_{i}=m\overset{k}{\underset{j=1}{\sum }}{\left(d_{j}^{i}\right)}^{n}$$

where *R_i_* is the *R* value of the half-month (MJ·mm·km^−2^·h^−1^); *k* is the number of days in the half-month *i*; and *d^i^_j_* is the effective precipitation for day *j* (i.e., ≥12 mm) of the half-month *i* [29]. The parameters *m* and *n* were calculated as follows:

*m* = 21.586 × *n*^−7.1891^ (3)

*n* = 0.8363 + 18.114/d_12_ + 24.455/y_12_ (4)

where d_12_ is the average daily rainfall (≥12 mm), and y_12_ is the yearly average rainfall for days with rainfall ≥12 mm. The annual *R* was firstly aggregated based on the *R* value of each half-month for each data point and then spatially interpolated for a continuous value surface using the Kriging method.

**Figure 4:**
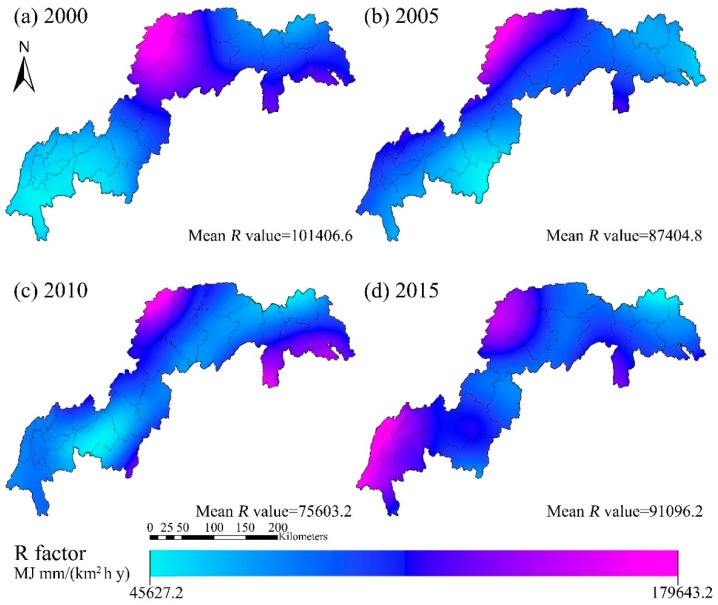
Estimated changes in the spatial pattern of rainfall erosivity, *R* factor (2000–2015).

The distributions of the annual *R* factor in the TGRR in 2000, 2005, 2010 and 2015 are shown in Figure 4. The highest and lowest *R* values (MJ·mm·km^−2^·h^−1^·y^−1^) averaged across the TGRR were 179,643.2 and 55,891.36 in 2000, 145,085.6 and 62,923.52 in 2005, 148,337.6 and 45,050.72 in 2010, and 134,880.48 and 53,813.92 in 2015, respectively. The lowest values of R (<64,000 MJ·mm·km^−2^·h^−1^·y^−1^) were mostly observed in the southwest part of the TGRR. The highest *R* value (>128,000 MJ·mm·km^−2^·h^−1^·y^−1^) was observed in the northern TGRR.

#### 3.1.2. Soil Erodibility Factor (*K*)

The soil erodibility factor, *K*, was estimated using the EPIC (Erosion/Productivity Impact Calculator) model of soil properties [30] which has also been applied in the National Soil and Water Conservation Survey of China [31]:

(5) $$K=0.1317\times \left(0.2+0.3\times e^{\left[-0.0256*San\times \left(1-\frac{Sil}{100}\right)\right]}\right)\;\times {\left(\frac{\mathrm{Sil}}{\mathrm{Cla}+\mathrm{Sil}}\right)}^{0.3}\times \left[1-\frac{0.25\times \mathrm{TOC}}{\mathrm{TOC}+e^{\left(3.72-2.95\times \mathrm{TOC}\right)}}\right]\times \left[1-\frac{0.7{\times \mathrm{SN}}_{1}}{{\mathrm{SH}}_{1}+e^{\left(22.9{\times \mathrm{SN}}_{1}-5.51\right)}}\right]$$

where *San* (%) is the sand content (0.05–2 mm); *Sil* (%) is the silt content (0.002–0.05 mm); *Cla* (%) is the clay content (<0.002 mm); *TOC* (%) is the soil total organic carbon content; and *SN_1_* = 1–*San*/100. Data retrieved from the Food and Agriculture Organization of the United Nations (FAO)’s HWSD soil database v1.2 (at 1 km spatial resolution) was used.

**Figure 5:**
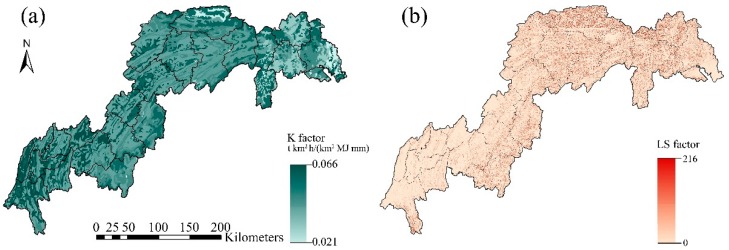
Estimated distributions of (**a**) soil erodibility, *K* factor; (**b**) slope length and steepness, *LS* factor.

Spatial pattern of the *K* value over the TGRR is presented in Figure 5a, with an averaged value of 0.035 t·km^2^·h·km^−2^·MJ^−1^·mm^−1^. The least erodible soils (*K* values < 0.029 t·km^2^·h·km^−2^·MJ^−1^·mm^−1^) are sandy soils (75%) mostly found in dry cropland areas. The most erodible soils (*K* values in the range of from 0.029 to 0.035 t·km^2^·h·km^−2^·MJ^−1^·mm^−1^) are with relatively greater slit content (45%) found in forests.

#### 3.1.3. Slope Length and Steepness Factor (*LS*)

The *LS* factor is a combination of the slope length factor (*L*), representing the ratio of soil loss in a place at a specific slope length, and the slope steepness factor (*S*), referring to the influence of slope gradient on erosion [21,32]. Following previous studies [33,34,35,36], the *LS* factor was calculated for each cell as:

L = (λ/22.13)^m^ (6)

(7) $$m=\frac{\beta }{1+\beta }$$

(8) $$\beta =\frac{\sin\theta /0.0896}{\left[3\times {\left(\sin\theta \right)}^{0.8}+0.56\right]}$$

where λ is the horizontal slope length; the exponent *m* is related to the ratio of rill to inter-rill erosion(*β*), and; *θ* is the slope angle in degree being also used to estimate slope steepness factor *S*:

(9) $$S\;=\{\begin{array}{c} 10.8\sin\theta \;+\;0.03,\;s\;<\;9\%\\ 16.8\sin\theta –0.5,\;s\;\geq \;9\% \end{array}$$

In this study, the *LS* factor was estimated based on a high-resolution (30 m) digital elevation model (DEM) using the System for the Automated Geoscientific Analyses (SAGA) software before being resampled onto 1 km spatial resolution using ArcGIS.

Distribution of the *LS* factor is mapped in Figure 5b, with an averaged value of 2.27 in the TGRR. Low LS values (<2.5) were found in the majority of the region, while high values (>42) mostly occurred in the northeast areas and coincided with the escarpments of the Wu Mountains, rendering these areas highly susceptible to soil erosion.

#### 3.1.4. Cover Management (*C*) and Control Practice (*P*) Factors

The cover management factor, *C*, represents the ratio of soil loss in an area under specific cover and management conditions. It addresses the combined effects of canopy cover, surface vegetation, surface roughness, prior land use, mulch cover and organic material below the soil surface [37]. Following Yang [3], the *C* factor was estimated based on NDVI: the equations were constructed based on correlations between NDVI and the *C* factor values obtained from RUSLE guidelines. Four sets of annual NDVI data covering the four time periods were extracted from the MODIS images.

The support practice factor, *P*, which reflects the effect of contouring and tillage practices [38], can be estimated based on land cover type [21]. In this study, land cover data at 30 m resolution were purchased from the Data Centre for Resources and Environmental Sciences at the Chinese Academy (RESDC) (http://www.resdc.cn). This product is based on visual interpretations of Landsat images guided by expert knowledge-based principles and quality control measures. The *p* values for different land cover types were derived from the exiting studies (Table 2).

**Figure 6:**
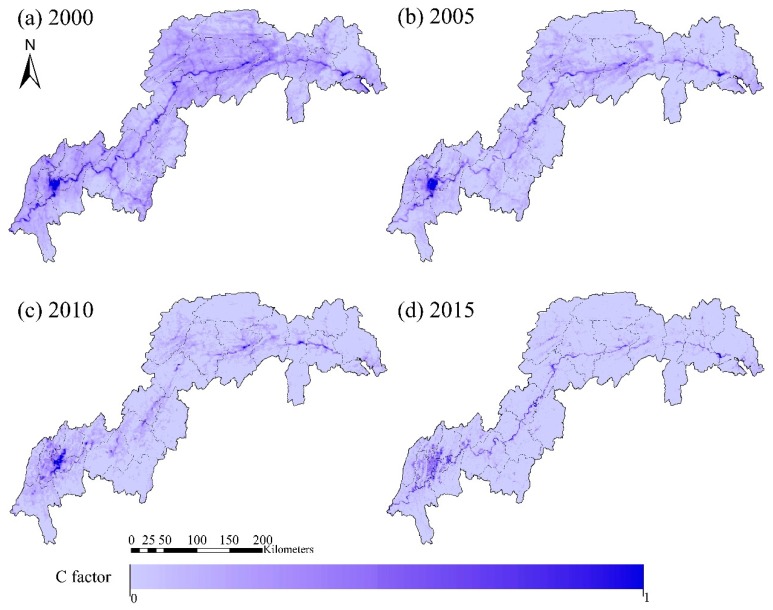
Estimated changes in the spatial pattern of cover management, *C* factor (2000–2015).

The estimated *C* factor in 2000, 2005, 2010 and 2015 are provided in Figure 6. High values of the *C* factor were found along the Yangtze riverway and in artificial areas, whereas low *C* values were found mostly in forest areas. The *C* factor in general decreased in the TGRR from 2000 to 2015, with the greatest reduction in Yuyang County from 0.7 to 0.13, as a consequence of NDVI improvement.

**Figure 7:**
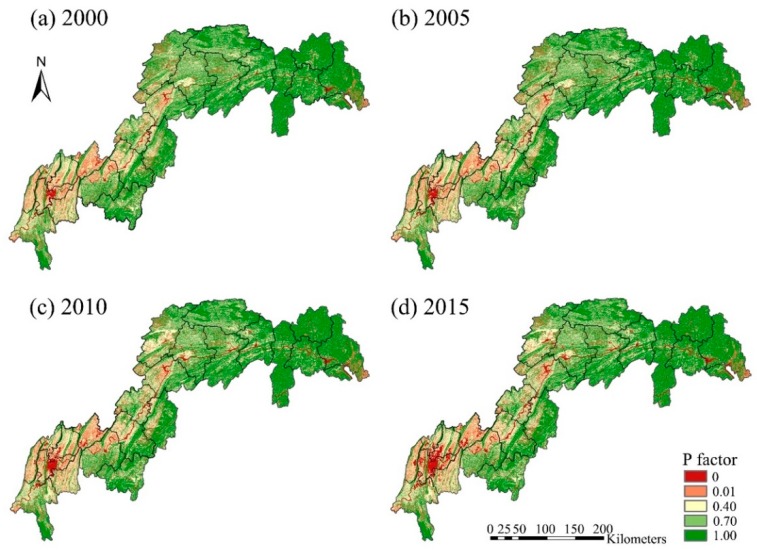
Estimated changes in the spatial pattern of support practice, *P* factor (2000–2015).

The changing patterns of *P* factor between 2000 and 2015 are provided in Figure 7. The changes were mainly driven by increased extents of woodland area (by ~690 km^2^) and artificial areas (by ~1080 km^2^), and by decreased extents of arable (by ~980 km^2^) and grassland areas (by ~1130 km^2^).

### 3.2. Changes in Land Cover Related to Soil Erosion

The Automatic Linear Modelling (ALM) procedure in IBM SPSS Statistics ver. 25 was applied [41] to identify the key land cover types whose changes are associated with the estimated soil erosion risk. The ALM is an improvement of the traditional linear regressive procedure and can provide data preparation and variable selection in an automatic manner. The changes (or the percentage increase/decrease) in the sixteen types of land cover between 2000 and 2015 were analysed using ALM for their importance in predicting the estimated changes in soil erosion risk. The importance of a variable refers to the change in residual sum of squares of the model when the variable is removed. The values of importance are normalised so that values of all variables sum up to one. This procedure could be implemented automatically with IBM SPSS. All variables were organised at the county and district levels, resulting in 23 observations. The method was used in its simplest form to produce a standard model, together with a forward stepwise method using the adjusted R-squared for model selection (variables inclusion/exclusion).

### 3.3. Interactions between Urbanisation and Natural Resource Management and Their Impacts on Soil Erosion

Urbanisation rate (urban fabric %) was calculated for the county and district level divisions. Impacts of natural resource management were considered from two perspectives: (i) vegetation diversity as calculated using the Shannon Index [42] for diversity of the nine arable, woodland and grassland types, and (ii) vegetation density as measured using the mean NDVI of the division. The Shannon Index was calculated as $-\overset{R}{\underset{i=1}{\sum }}p_{i}\ln p_{i}$, where $p_{i}$ is the proportion of the vegetated land cover belonging to the *i*th types in the dataset. Changes between 2000 and 2015 in the above two indicators were prepared as explanatory variable of the estimated changes in soil erosion. The Generalized Linear Model (GLM) procedure in the IBM SPSS statistics was used to analyse the potential effects of the variable interactions. A full model was built first with all individual and interactions among the three explanatory variables as factors. Then, by removing the least significant factor (with the greatest *p* value) at one time, we rebuilt the model until the remaining factor were all significant at 0.05 level.

## 4. Results

### 4.1. Estimated Rate of Soil Erosion by Water

**Figure 8:**
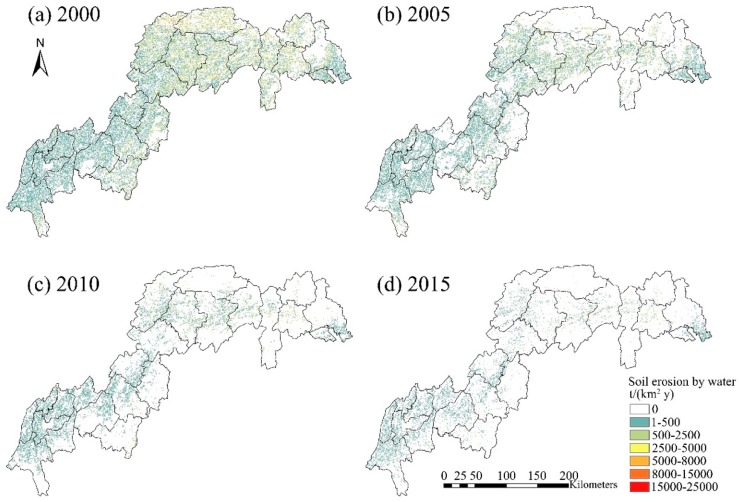
Spatial patterns of annual soil erosion by water in the Three Gorges Reservoir Region in 2000, 2005, 2010 and 2015.

The resulting soil erosion actual maps are shown in Figure 8. The estimated soil loss actual averaged across the TGRR (t·km^−2^·y^−1^) were 250.17 (2000), 91.61 (2005), 37.99 (2010) and 18.74 (2015), showing a declining trend. The cell-level estimations were further classified into the six grades of erosion established by the Ministry of Water Resources of China (SL190-2007). Extents and proportions of areas at different erosion grades are summarised for the four time periods (Table 3). Areas eroded at all grades higher than grade 1 (slight erosion) were estimated to decrease from 2000 to 2015. As of 2015, the TGRR was estimated to be free of the areas eroded in extremely intense or severe ways (grades 5 and 6) at the scale this study concerned. The remaining intensely eroded areas (0.005% of the region in 2015) mainly distributed in southeast TGRR, i.e., Fengjie, Wushan and Badong (Figure 8d).

### 4.2. Land Cover Determinants of Soil Erosion in the TGRR

The best model generated from the Automatic Linear Modelling (ALM) procedure had an adjusted R-squared value of 0.739, indicating a good fitness of using changes in land cover types as explanatory factors in the linear modelling of soil erosion change. The changes in land cover types for different administrative divisions are provided in Table S1. The land cover types found significant (*p*-value < 0.05) are listed in Table 4 with river having the greatest relative importance. Changes in river surface was found negatively associated with soil erosion risk, meaning the soil erosion risk decreased as the water level rose. Other land cover types found important include sparse grassland, other woodland (mixed, very sparse woodland and orchards) and shrubs, which were all positively associated with soil erosion.

### 4.3. Effects of NDVI and Vegetation Diversity Varied at Different Urbanisation Rates

**Figure 9:**
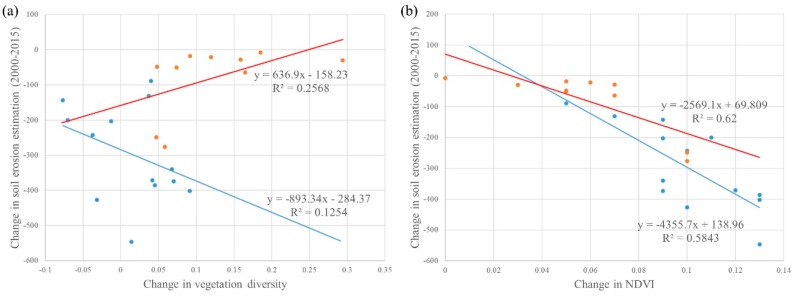
Effects of the interaction (**a**) between urbanisation and vegetation diversity and (**b**) between urbanisation and NDVI on soil erosion risk. The blue and red lines indicate that the increase in urbanisation rate was lower and higher than 0.1, respectively.

The final outputs from the Generalised Linear Model procedure includes two significant (*p*-value < 0.05) interactions that influenced changes in soil erosion: (i) between in NDVI and urbanisation rate and (ii) between vegetation diversity and urbanisation rate (Table 5). We further classified the data into two groups, according to whether the division’s urbanisation rate had increased greater than 0.1 (from 2000 to 2015) or not. The vegetation diversification (increased Shannon Index) was associated with (i) decreased soil erosion in the divisions with lower increase in urbanisation rate, and (ii) increased soil erosion when the divisions had greater increase in urbanisation rate. The improvement of NDVI was found to have a greater impact on soil erosion reduction when the divisions have a lower urbanisation rate (Figure 9b).

## 5. Discussion

The present study provides a large-scale assessment on the spatio-temporal changes in soil erosion risk in the Three Gorges Reservoir Region (TGRR) which has experienced rapid and complex land use/cover change driven by multi-sectoral policies, e.g., to accommodate population migrated due the Three Gorges Project, pursue urban and rural development, and protect natural resources.

Among the 23 administrative divisions focused in this study, eight divisions (Fuling, Shizhongxin, Banan, Beibei, Bishan, Changshou, Jiangjin, Yubei) had relatively low soil erosion estimates in 2000, ranging from 31 to 94 t·km^−2^·y^−1^. As of 2015, averaged estimated soil erosion in these divisions decreased by approximately 60%, mainly due the observed decreases in rainfall-based R and land cover-driven *C* factors. Only small changes in the NDVI-based *P* factor between 2000 and 2015 was found, as these divisions are mainly urban areas. The other fifteen divisions had relatively higher estimated soil erosion risk, i.e., from 107 to 548 t·km^−2^·y^−1^, in 2000. The decrease in the averaged soil erosion risk was found to be almost 90 percent by 2015. Such a sharp decrease was mainly driven by the *C* factor which decreased significantly as a consequence of improved NDVI, and which overran the effects of the increased *R* and *P* factors. The improvement of NDVI in China was found to be associated with successful implementation of reforestation policies [43] and dense forests are known to provide service of runoff reduction [4] and soil erosion mitigation [44,45]. At the cell-level, areas of intensive and severer soil erosion grades (>2500 t·km^−2^·y^−1^) should be given high priority in conservation management. In 2015, these areas covered only 0.005% of the TGRR, however, contributed to 10.1% of the total regional soil erosion risk (1.10 million ton).

River surface area is identified as a key land cover type associated with soil erosion risk in the TGRR. The general increase in river surface between 2000 and 2015 was due to the construction of the Three Georges Dam and the consequent water level rose. The majority of the population previously lived by the river side in the rural TGRR were resettled and most of them (1.3 out of 14.8 million) [46] were migrated to regions outside the TGRR. It seems that an increased river surface could be linked to a reduced level of local human activities and, thus, lowered anthropogenic driving force of soil erosion. Moreover, previous ground soils susceptible to high erosion risk in the valley area were submerged and excluded from the estimations for the later years. This might also contribute to the estimated reducing trend of soil erosion risk.

Previous studies on estimating the soil erosion in the TGRR mainly focused on small watersheds, for example in Kai and Zigui counties, Xinzheng Village and Lianghe Village [47,48,49]. These small watersheds are usually dozens of hectares in size and their estimated soil erosion ranged from 306 to 9452 t·km^−2^·y^−1^, which are higher than our large-scale estimations, for example, 250.17 in 2000 and 18.74 t·km^−2^·y^−1^ in 2015, owing to the fact that the lower level spatial heterogeneity has not been captured due to data limitations. Our estimations are in good agreement with the limited previous large-scale studies on soil erosion risk in the TGRR. Cai et al. [18] evaluated soil erosion risk in the sub-division of TGRR in Hubei Province and found areas of light and slight soil erosion grades (<2500 t·km^−2^·y^−1^) took up 96.27% of total area which is close to our estimation of 97.72%. Li et al. [10] reported that counties suffered the highest soil erosion risk in Chongqing Municipality were Fengjie, Wushan and Badong, which are also among the divisions of the greatest soil erosions risk estimated in our study. The differences between our study and these two studies are mainly due to the different data sources used, especially for calculation of the *R* and *K* factors. Compared to the daily precipitation data used in our study, Li et al. [10] estimated the *R* factor based on the monthly precipitation data from Chongqing Municipal Meteorological Bureau. Due to the lack of local data, Cai et al. [18] calculated the *K* factor in Hubei Province based on a method initially proposed based on the soil conditions in the United States. While in the present study, a modified method by Zhang, et al. [50] was used and this method is also used in the National Soil and Water Conservation Survey of China. Our results are also comparable to those conducted in areas outside the TGRR. For example, Zhang et al. [2] studied soil erosion variability in Shanxi Province, on the Loess Plateau of China. They observed high soil erosion rate in areas with high terrain alteration, high slopes, and land with sparse vegetation. In the Bohai Rim (China), Xu et al. [40] claimed that vegetation coverage and soil erosion control practices are important factors for future soil conservation.

The temporal window of this study (from 2000 to 2015) covers the three periods concerned by the China’s National Tenth (2001–2005), Eleventh (2006–2010) and Twelfth (2011–2015) Five-Year Plans. The reductions in average soil erosion over these three five-year periods were 9.3, 3.2 and 1.1 million tons, respectively. In the white book of the Tenth Five-Year Plan, three chapters on saving resources and protecting environment are put under the theme “Population, resources and environment”, with one chapter on urbanisation plan. In the Eleventh Five-Year, five chapters on natural resource management and ecological restoration are organized under the theme “Building a resource-conservative and environment-friendly society”, with three chapters on regional development. In the Twelfth Five-Year, there are six chapters on responding to climate change, developing circular economy and promoting ecological protection under the theme “Green development”, and four chapters planning rural development. The increasing focus on sustainable environment and urbanisation in the recent Five-year Plans shows the determination of the Chinese government to improve environment quality, restore ecosystem and modernise urban and rural developments. It is within this context that not only the TGRR, but also some other regions in China as previously discussed are benefited from conservation actions at different administrative levels.

Controlling soil erosion may reflect on how land-use and land-cover patterns are managed and be influenced by policies targeting different sectors. From this aspect, our analysis on the interactions between urbanisation and natural resources management is particularly useful, as it provides more in-depth information, based on which policy implications could be drawn. For example, urban green infrastructure design should take into consideration the likely impact of vegetation diversity on amplifying soil erosion in highly urbanised area. Besides, planting forests which can typically contribute to greater NDVI could help mitigating soil erosion risks in both urban and rural area.

## 6. Conclusions

This study produced timely estimates of recent soil erosion actual change between 2000 and 2015 over the whole of the TGRR. The estimation by the RULSE model showed that the average soil erosion by water in the TGRR is 18.74 t·km^−2^·y^−1^ in 2015, which equates to a total soil loss of 1.10 × 10^6^ t annually and a decrease of 13.6 × 10^6^ t from 2000. Areas that suffer from severe soil erosion are estimated in the middle and northern TGRR, in particular, in the Wu Mountains. Soil erosion risk decreased as the water level rose and river surface increased. Two interactions (between NDVI and urbanisation rate, and between vegetation diversity and urbanisation rate) were found to influence in soil erosion actual. The RUSLE method is useful tool to characterise long-term changes in soil erosion actual over a large area. The approaches used in our study are transferable to other areas exhibiting similar socio-environmental conditions. Our results could provide useful information for the development of integrated urbanisation and natural resource management policy on regional soil conservation. Future investigations are encouraged to examine the seasonal dynamics of the TGRR’s actual soil erosion risk due to the periodic water level change, as the total water volume is managed and varies temporally.

## Figures and Tables

**Table 1:** List of the datasets used in the RUSLE model and their sources.

Type	Environmental Variables	Resolution	Source
Terrain	DEM	30 m	SRTM digital evaluation (NASA)
	Slope	30 m	SRTM digital evaluation (NASA)
Climate	Daily rainfall from 2000 to 2015	-	Local meteorological stations
Vegetation	NDVI from 2000 to 2015	250 m	MODIS images
Land	Land use/cover type (LUCC) at 2000, 2005, 2010 and 2015	30 m	Resources and Environment Data Cloud Platform, Chinese Academy of Science
Soil property	Soil type	1 km	HWSD soil database v1.2 (FAO)
	Sand	1 km	HWSD soil database v1.2 (FAO)
	Silt	1 km	HWSD soil database v1.2 (FAO)
	Clay	1 km	HWSD soil database v1.2 (FAO)
	TOC	1 km	HWSD soil database v1.2 (FAO)

**Table 2:** The *P* factor values of different land use types on the TGRR.

Land Use Type	*p* Value	Reference
Paddy fields	0.01	[39]
Dry cropland	0.4	[40]
Dense forest	1	[40]
Shrub	1	[40]
Sparse forest	1	[40]
Other woodland	0.7	[2]
Dense grassland	1	[2]
Moderate dense grassland	1	[2]
Sparse grassland	1	[2]
River	0	[2]
Lake	0	[2]
Reservoir	0	[2]
Mudflat	0	[2]
Urban fabric	0	[40]
Rural fabric	0	[39]
Construction and transportation units	0	[2]

**Table 3:** Changing soil erosion grades in the Three Gorges Reservoir Region between 2000 and 2015. The soil grade classification accords to the Ministry of Water Resources of China (SL190-2007).

Soil Erosion Rate (t·km^−2^·y^−1^)	Erosion Grade	2000		2005		2010		2015	
		Extent (km^2^)	Proportion (%)	Extent (km^2^)	Proportion (%)	Extent (km^2^)	Proportion (%)	Extent (km^2^)	Proportion (%)
<500	Grade 1 (slight)	45,507	77.54	50,057	85.25	54,064	92.08	58,051	98.871
500–2500	Grade 2 (light)	2985	5.09	3045	5.19	1588	2.7	632	1.076
2500–5000	Grade 3 (moderate)	2798	4.77	1622	2.76	1039	1.77	28	0.048
5000–8000	Grade 4 (intense)	2715	4.63	1509	2.57	710	1.21	3	0.005
8000–15,000	Grade 5 (extremely intense)	2478	4.22	1327	2.26	694	1.18	0	0
>15,000	Grade 6 (severe)	2203	3.75	1156	1.97	619	1.06	0	0

**Table 4:** Importance of changes in land cover types on soil erosion.

Land Cover Type	Coefficient	Standard Deviation	T	*p*-Value	95% Confidence Interval		Importance
					Low Limit	Upper Limit	
River	−6.199	1.482	−4.181	0.001	−9.378	−3.019	0.308
Sparse grassland	17.201	4.595	3.743	0.002	7.346	27.057	0.247
Other woodland	6.860	2.512	2.730	0.016	1.471	12.248	0.131
Shrub	1.977	0.756	2.614	0.020	0.355	3.598	0.120

**Table 5:** Parameter estimation of Generalized Linear Model.

Variables	B	Standard Deviation	95% Confidence Interval		*p*-Value
			Low Limit	Upper LIMIT	
Intercept	167.232	53.2636	62.837	271.627	0.002
NDVI	–4817.538	552.0762	–5899.588	–3735.489	0.000
NDVI × Urban rate	39,743.093	11,681.8554	16,847.077	62,639.109	0.001
Urban rate × Vegetation diversity	–5113.203	1804.2045	–8649.379	–1577.027	0.005

## Supplementary Materials

The following are available online at https://www.mdpi.com/1660-4601/16/10/1856/s1, Table S1: Land cover changes between 2000 and 2015 in the Three Gorges Reservoir Region (in km^2^).
