# Supplementary material for: The Implication of Land-Use/Land-Cover Change for the Declining Soil Erosion Risk in the Three Gorges Reservoir Region, China
Source: Int J Environ Res Public Health. 2019 May 26;16(10):1856. doi: 10.3390/ijerph16101856 (PMC6572475; doi:10.3390/ijerph16101856)
Supplement: Supplementary file 1 [file ijerph-16-01856-s001.pdf]

Table 1. Land cover changes between 2000 and 2015 in the Three Gorges Reservoir Region (in km^2).

Division	Paddy fields	Dry cropland	Dense forest	Shrub	Sparse forest	Other woodland	Dense grassland	Moderate dense grassland	Sparse grassland	River	Lake	Reservoir	Mudflat	Urban fabric	Rural fabric	Construction /transportation
Fengjie	−5.2	−23	2.6	−77.1	−15.1	87.3	−6.2	−18.2	−1.5	34	−0.1	2.1	−0.2	6.1	4	9.2
Kai	−5.1	308.3	9.3	17.4	14.9	−10.5	15.5	−397	−1.1	14.3	0	12.7	−3.9	5.2	5.1	16.6
Wanzhou	−24.2	−43.6	27.5	21.2	−8.2	5.3	−24.3	−18.9	−0.1	36.5	0	4.5	−12.4	12.4	4.1	20
Wushan	−0.5	−19.7	−0.8	−9.1	7.5	7	14.7	−33.2	−9.9	20.1	−0.2	12.6	−0.1	2.6	4.9	5.4
Wuxi	−0.7	−31.3	12.7	−9.7	15.6	0.6	6	23.8	−29.5	0.8	0	2.4	0.1	3	1.3	6.6
Yunyang	19.6	−106.7	4.3	10.1	−8.6	−8.6	12.5	−20.3	1.7	73.1	0	3.7	−9.4	7	6.5	14.8
Zhong	−16.8	−18	4.7	0.8	2.2	11.8	−1.9	−9.5	−0.5	26.5	0.1	−0.2	−11.4	3.1	3.8	6
Shizhu	−28.5	−71.7	151.3	35.5	6.6	4.3	−16.5	−98.3	−14.8	4.5	0.1	5.2	−1.8	4	4.6	15.1
Fengdu	1.1	−48.8	175.1	84.5	−3.6	4.9	7.4	−246.8	−0.4	10.1	0	1.8	−2	1	1.8	14.3
Wulong	8.9	−28.5	2.8	148	35	0.5	39.1	−222.4	−8	0.3	0	8.1	0	0.6	3.6	11
Fuling	−10	−39.5	49.2	42.4	−24.5	14.5	16.8	−102.7	−0.1	16.4	−0.1	0.1	−11.1	7.7	4.5	36.7
City centre of Chongqing	−144.7	−139.3	2.7	3.1	−13.3	−3.3	2.4	0.3	−0.2	6.1	−0.1	−0.4	−2.6	90.6	25.2	173.7
Banan	−20.5	−28.6	−24.8	4.6	16.5	2.1	1.7	−1.6	−0.1	9.2	0	−0.3	−8.5	13.5	-0.1	36.9
Beibei	−27	−25.1	4.5	−6	−0.8	0.4	−0.2	−0.2	0.1	0.8	−0.1	0.1	0	3.9	7.3	43.2
Bishan	−20.5	−15.7	−0.1	0.5	0.9	0.1	0.4	−0.1	0.3	0	0	1.2	0	12.5	1.5	19
Changshou	−59.9	−13	−23.3	8.1	15.5	7.3	0.8	0	0	3	0.4	−0.6	−2.8	35.5	4.7	26.2
Jiangjin	−13	54	1.5	−7.4	−101.1	−6.6	1.2	0.1	−0.4	−0.6	0.1	0	−0.1	6.4	4.6	59.8
Yubei	−90.6	−105.3	8.8	−5.9	−4.3	0.5	0	−2.4	0.1	1.9	0	1.4	−1.8	68.6	-0.1	129.8
Zigui	−11.2	−19.6	−16.8	1.4	−11.7	2	−4.6	2.5	1.1	45.9	0	1.5	−0.9	6.2	0.9	3.4
Dianjun	−7.6	−6.2	−4.3	−5	9.8	0.7	−0.1	0	0	1.4	0	0	−0.4	1.6	0.6	9.9
Xingshan	−2.2	−60.6	−1.5	−5.1	49.8	7.6	1.3	0.6	0	5.3	0	0.2	−0.1	2.8	0.4	1.4
Yiling	−2.4	−19.4	−28.1	−21	10.3	18.3	5.5	0.3	0	12.6	−0.3	−0.6	−0.9	0.7	-0.5	25.3
Badong	−9.7	−5.8	−33.1	8.5	−18.5	2.7	15.8	−16	2.6	37.3	0	1.7	0.5	3.7	1.6	8.8
